# Mainly high phenotypic stability of black spruce clones for growth and wood traits in contrasted environments within the current breeding zones and multitrait selection in Québec's seed and breeding zones

**DOI:** 10.1093/g3journal/jkaf120

**Published:** 2025-05-30

**Authors:** Mireille Desponts, Josianne DeBlois, Guillaume Otis-Prud’homme, Martin Perron

**Affiliations:** Direction de la recherche forestière, Ministère des Ressources naturelles et des Forêts, 2700, rue Einstein, Québec, QC, Canada G1P 3W8; Direction de la recherche forestière, Ministère des Ressources naturelles et des Forêts, 2700, rue Einstein, Québec, QC, Canada G1P 3W8; Direction de la recherche forestière, Ministère des Ressources naturelles et des Forêts, 2700, rue Einstein, Québec, QC, Canada G1P 3W8; Direction de la recherche forestière, Ministère des Ressources naturelles et des Forêts, 2700, rue Einstein, Québec, QC, Canada G1P 3W8

**Keywords:** advanced breeding population, clonal test, genotype × environment interaction, genotypic value, genotypic ecovalence value, *Picea mariana*

## Abstract

Selected trees from various progeny trials cannot be directly compared. As parents of clonal seed orchards and components of the next breeding cycle, it is crucial to verify their stability in different soil and climatic conditions and to rank them for multiple traits. A subset of the best trees selected for height at 10 or 15 years was cloned to establish 2 clone trials per population for 4 breeding populations, covering different bioclimatic domains of Québec. Between 80 and 119 clones per population were compared for height, diameter at breast height, indirect measure of wood density (Pilodyn 6J Forest device) and acoustic velocity (Fiber-gen Hitman ST300). Linear mixed models were used to estimate various genetic parameters, including genotypic values, using the Best Linear Unbiased Prediction (BLUP) method. With the genotypic values at 15 or 16 years for acoustic velocity and height, a selection index was calculated for ranking the clones. Clonal variances are significant for all growth and wood traits. Clonal heritabilities are low for growth traits with 1 exception (0.11–0.30) and range mainly from moderate to high for wood traits (0.29–0.70). Genotype × environment (G × E) interactions for growth traits are low for 2 populations (0.87–1) and mainly moderate for the 2 others (0.53–0.92). For wood traits, G × E interactions are low to almost nil and are mostly moderate for 1 population (0.69–1). In general, clones exhibit high stability (2 BLUP-based stability indexes) for growth and wood traits in contrasting soil and climatic conditions, except for the growth traits of 2 populations.

## Introduction

Black spruce [*Picea mariana* (Mill.) B.S.P.] is a dominant species in the boreal forest of North America. Its distribution over a vast area indicates its adaptation to varied climate and pedoclimatic conditions (e.g. Beaulieu *et al*. 1989; Pedlar *et al*. 2021).

A black spruce genetic tree improvement program has been undertaken by the Government of Québec, with the goal of increasing yield and quality of the tree stem in plantations (e.g. Vallée 1975). With the help of Natural Resources Canada, 5 seeds (deployment) and breeding zones were previously delineated based on 10-year measurements from 4 provenance trials planted in 1974–1975 (Beaulieu *et al*. 1989).

As with most tree breeding programs, seedlings were used for progeny trials and consequently for selecting superior trees as future parents (e.g. White *et al*. 2007). This means that all individual black spruce progeny trees were tested in only 1 environment, and moreover many top-ranked families were not compared since they were in different progeny trials series linked to different seedling seed orchards, even within a specific seed and breeding zone. Therefore, with seedlings, there is no replication of the trees, making their ranking less precise compared to ranking using clone trials (e.g. Isik *et al*. 2004), even with advanced quantitative genetics methods (individual tree analysis, spatial error modeling, etc.) (e.g. Costa e Silva *et al*. 2013).

Clone trials primarily allow the identification of (1) the best genotypes across all sites for evaluated traits and (2) superior clones that maintain similar ranks in different environments (Ledig and Kitzmiller 1992; Mullin and Park 1994; Baltunis *et al*. 2013). Indeed, phenotypic stability, which has been studied in spruce species (e.g. Isik and Kleinschmit 2003; Wahid *et al*. 2012; Skrøppa and Steffenrem 2021), is important as it ensures consistent performance of clones relative to the average performance at the site in changing environments. This characterizes phenotypic plasticity, which, in the context of climate change and environmental instability, is crucial information in breeding programs. Climate change and advancements in tree breeding programs have increased the need to estimate genotypic parameters and to assess phenotypic stability at the clonal level. Therefore, large-scale tree breeding programs now take greater account of phenotypic stability, aiming to produce trees that are more resilient and better adapted to changing conditions.

Using open-pollinated families, Perron *et al*. (2013) estimated very little genotype × environment (G × E) interaction for height growth and wood density, as well as for several wood traits (Desponts *et al*. 2017). However, this adaptability remains to be verified with clones, expected to have a narrower range of optimal conditions and likely more significant G × E interactions. A low clone × site interaction for the entire population would indicate comparable performance across the intended use area and validate the 5 existing seed and breeding zones for the next breeding cycle and clonal seed orchards (SO-2). Moreover, highly stable clones, individually considered in different climates and pedoclimatic conditions, would represent an adaptive advantage in the context of climate change and unstable environmental conditions. This adaptive response capability in various environments, and the ability for some to maintain their relative superiority, could be interesting selection traits for developing the next generation of improved trees (Sonesson and Eriksson 2000; Mátyás 2006; Baltunis *et al*. 2013; Joyce and Rehfeldt 2017). Actually, most large-scale breeding programs for conifers include multitrait information on genetic parameters and genetic by environmental interaction (G × E) at the family level, but much less information at the clonal level.

Up to now, the Québec's black spruce breeding program has not yet largely used multitrait selection and no direct evaluation of wood quality traits, primarily due to the need to measure a large number of trees for genetic parameter estimation and selection, as well as logistical difficulties in multiple wood quality trait measurements (e.g. Perron *et al*. 2013). Moreover, indirect sampling methods for wood quality traits, applicable to a large number of trees (e.g. Chen *et al.* 2015; Fundova *et al*. 2019), are fortunately feasible for the important trait of wood stiffness, especially expressed by the dynamic modulus of elasticity (MoE) (e.g. Desponts *et al*. 2017).

The main objective of this study on clone trials is to better characterize the parents of various Québec's seed and breeding zones to rapidly enhance the qualities of black spruce trees in plantations, particularly wood stiffness and environmental stability of growth. This objective concerns the best-ranked selected trees (STs) based on height breeding value among the progeny trials (1982–1993), as they are present in breeding populations, clonal seed orchards, and their associated clone trials (Table 2; Desponts and Numainville 2013). The secondary objectives of this study include (1) estimating genotypic parameters for growth and wood traits, (2) ranking clones using genotypic values and a multitrait selection index (SI) to quickly increase anticipated genetic gains for the next breeding cycle (more crosses with the best-ranked ST) and production populations (SO-2), (3) evaluating the phenotypic stability of clones with 2 Best Linear Unbiased Prediction (BLUP)-based stability indexes, and finally (4) validating the current breeding zones for ST. We present the measurement results for growth, wood density, and 2 indirect wood stiffness traits (acoustic velocity and dynamic MoE) from 15- or 16-year-old black spruce clonal trials representing 3 seed and breeding zones out of 5.

## Materials and methods

### Biological material

**Fig. 1.**
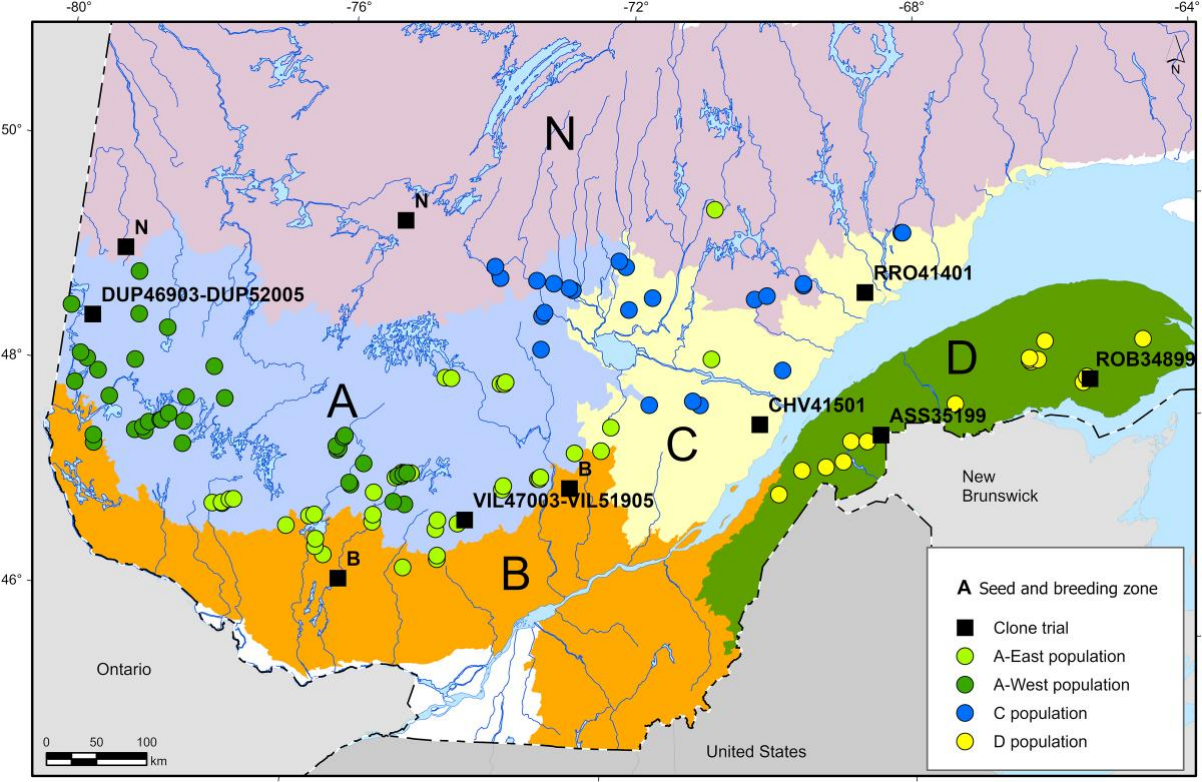
Geographical distribution of ancestral provenances of black spruce parental individuals for the 4 study clonal populations in zones A, C, and D, as well as clone trial locations for all Québec's seed and breeding zones.

The study involves 4 distinct clonal populations representing 3 breeding zones, covering different bioclimatic subdomains of southern Québec (MRNF 2022), and each population was evaluated in 2 clone trials within their current seed and breeding zone (Fig. 1; Table 1). Each clonal population consists of a subset of STs and is associated with 1 or 2 clonal seed orchards (SO-2), according to seed requirement in their respective seed and breeding zone (Table 1; Desponts and Numainville 2013). Depending on the zone, a clonal population varies from 80 to 119 clones common to both clone trials, representing 46–67 families (Fig. 1; Supplementary Table 1). The clones were produced from cuttings taken from the ST, and the trials were established when the rooted cuttings were 3 years old. The clonal populations are a subset of 1,200 STs (5 breeding zones) that were chosen from over 540,000 candidates trees representing 5,750 open-pollinated families and tested in 42 progeny trials (1982–1993). They were retained based on a sequential selection: first retaining the best-ranked families and then choosing the best trees within those families. In addition to the height growth breeding value, the within-family selection included a phenotypic selection for form (stem and crown) and the absence of disease (Table 1; Desponts and Numainville 2013).

**Table 1.** Location of clone trials and climate characteristics*^a^* in their respective ecological districts.

Seed and breeding zone	Side-by-side A-West and A-East populations		C		D	
Clone trials	Duparquet (DUP46903 and DUP52005)	Villiers (VIL47003 and VIL51905)	Chauveau (CHV41501)	Rivière-aux-Rosiers (RRO41401)	Robidoux (ROB34899)	Asselin (ASS35199)
Ecological subregion*^b^*	5a-T	4c-T	5e-T	5g-T	5h-T	4f-T
Latitude (°N)	48° 27ʹ	46° 59ʹ	47° 58ʹ	49° 10ʹ	48° 21ʹ	47° 53ʹ
Longitude (°W)	79° 09ʹ	73° 55ʹ	70° 04ʹ	68° 40ʹ	65° 37ʹ	68° 27ʹ
Altitude (m)	298	456	238	232	265	336
Degree days > 5°C	1,407	1,359	1,401	1,316	1,306	1,435
Total annual precipitations (mm)	955.5	999.6	987.5	1,008.9	1,131.1	1,121.0
Annual mean temperature (°C)	1.4	2.0	2.4	1.7	2.7	2.3
Growing season (days)	145	140	160	156	157	152
Soil	Heavy clay	Silty sand	Sand	Sandy loam	Loam	Loam

^*a*^Data source: Régnière *et al*. (2017).

^*b*^ Saucier *et al*. (2003).

### Study trials

The 8 clone trials of the present study were established according to a randomized complete block design, each with 10 blocks and 2-tree plots for each clone. The 2 clone trials per clonal population are located within their respective seed and breeding zones (Fig. 1), but in sites with contrasting climates and pedoclimatic conditions (Table 1). It was thus possible to evaluate, within the same sites, trees (genotypes) from several progeny tests generally representing distinct populations within a breeding zone (see, for example, the 6 populations linked to the 6 seedling orchards and distributed across 11 progeny trials for zone A—Desponts and Numainville, 2013).

The first breeding zone concerned is zone D, located south of the St. Lawrence River, in the eastern yellow birch (*Betula alleghaniensis* Britt.)-balsam fir [*Abies balsamea* (L.)] bioclimatic domain, including a more boreal mountainous region of the eastern paper birch (*Betula papyrifera* Mash.)-balsam fir bioclimatic domain. The bedrock is mainly composed of limestone, shale, and sandstone. The Robidoux test (ROB34899) is located in the mountainous Chic-Chocs region, with rugged terrain, while the Asselin test (ASS35199), further south (Fig. 1), on hilly terrain. Both sites, established in 1999, are on till deposits, and the climatic conditions are comparable (Table 1).

The second zone, north of the St. Lawrence River, mainly consists of the continuous boreal forest, with the eastern paper birch-balsam fir bioclimatic domain and the spruce-moss bioclimatic domain (zone C). In zone C, the Rivière-aux-Rosiers (RRO41401) and Chauveau (CHV41501) tests, established in 2001, are located in the foothills of the Canadian Shield, the former on a thin till deposit, and the latter on a sandy deposit. Climatic conditions are harsher at Rivière-aux-Rosiers (Table 1), with a lower average annual temperature and a shorter growing season compared to the Chauveau site.

The last breeding zone covers the entire western paper birch-balsam fir and yellow birch-balsam fir bioclimatic domains (zone A). These 2 bioclimatic domains are also on the Canadian Shield with a bedrock of crystalline rocks and more acidic soils. However, the western sector of zone A, located in the Clay Belt, is characterized by a substrate mainly composed of glaciolacustrine clay deposits. In zone A, 2 distinct clonal populations were established side-by-side at 2-year intervals in 2 contrasting sites (Fig. 1), for a total of 4 clonal trials on 2 sites (Fig. 1; Table 1). One population (Villiers and Duparquet tests established in 2003) consists of STs from families from the center and east of the zone (hereafter A-East population), and the second population of STs from families mainly from central and western provenances (hereafter A-West population, Villiers and Duparquet tests established in 2005). The Villiers tests (VIL47003 and VIL51905) are situated in a hilly landscape on a till deposit, while the Duparquet tests (DUP46903 and DUP52005) are in a landscape with very little relief on a clay deposit. The climate at Duparquet is more continental, with lower average temperatures and precipitations compared to Villiers (Table 1).

### Data collection

Total height (TH) growth was measured at 5 and 10 years after the establishment of the clone trials. Then, at 15 years (16 years for zone C), all trees were measured for TH and diameter at breast height (DBH, at 1.3 m), while 1 ramet per plot, chosen based on trunk straightness and regular trunk shape, was subjected to wood quality measurements. In each series of 2 clone trials, a total of 1,122–2,731 trees were measured for growth, averaging 12–31 ramets per clone (Supplementary Table 1). Wood density was assessed by measuring the penetration depth of the Pilodyn 6J Forest device ($D_{\;pil}$; Proceq, Switzerland) at breast height. A steel rod was projected into the trunk with constant pressure, and the penetration depth was measured. The measurement was taken 2 or 3 times, or until 2 identical measurements were obtained (with the accuracy of 1.0 mm). Two indirect traits of wood stiffness were considered: (1) the propagation of an acoustic wave and (2) a dynamic MoE. Probes were installed 1 m apart along the trunk at a height of 1.3 m. Six measurements of the acoustic wave propagation time were taken (Fiber-gen Hitman ST300) and converted into velocity. The average value of these acoustic measurements ($V_{dir}$, wave propagation velocity in km · s^−1^) and the average penetration depth of the Pilodyn ($D_{\;pil}$, mm) were used to calculate an indirect measure of the MoE ($\mathrm{Mo}E_{dir+pil}$, GPa) for each tree using the equation of Chen *et al*. (2015):


$$\mathrm{Mo}E_{dir+pil}=\left(\frac{1}{D_{\;pil}}\right)\times 10\times \;V_{dir}^{2}.$$

Wood characteristics were measured on 572–1,441 trees (7–12 ramets per clone on average) depending on the seed and breeding zone. It should be noted that the number of ramets studied in the western population of zone A (VIL51905 and DUP52005) is significantly lower due to mortality of the cuttings during the first year after planting, attributed to drought and high temperatures during the summer of 2005.

### Models

For each breeding zone, an analysis of variance was performed for each trait of interest using the MIXED procedure of the SAS software package (version 9.4; SAS Institute, Inc., Cary, NC, USA) and the following linear mixed model:


$$Y_{ijkl}=\;\mu +\;S_{i}+\;B(S{)}_{\;j(i)}+\;C_{k}+\;(S\times C{)}_{ik}+(B(S)\times C{)}_{\;j(i)k}+\;\varepsilon_{ijkl},$$

where $Y_{ijkl}$ is the measured trait value of ramet *l* of clone *k* located in block *j* of site *i*, *μ* is the overall mean, $S_{i}$ is the fixed effect of site *i* (*i* = ROB34899, ASS35199, RRO41401, CHV41501, VIL47003, DUP46903, VIL51905, DUP52005), $B(S{)}_{\;j(i)}$ is the fixed effect of block *j* in site *i* (*j* = 1–10), $C_{k}$ is the random effect of clone *k* [*k* = 1–80 (zone A-West), 88 (zone C), 98 (zone D), or 119 (zone A-East), $C_{k}\;∼\;N(0,\;\sigma_{C}^{2})$], $(S\times C{)}_{ik}$ is the random effect of the interaction between site *i* and clone $k\;({\left(S\times C\right)}_{ik}\;∼\;N(0,\;\sigma_{S\times C}^{2}))$, $(B(S)\times C{)}_{\;j(i)k}$ represents the random effect of the clone by block interaction, which here corresponds to the experimental error (plot effect, $\left(B(S)\times C{)}_{\;j(i)k}\;∼\;N(0,\;\sigma_{B(S)\times C}^{2})\right)$, and $\varepsilon_{ijkl}$ is the residual error (within-plot error, $\varepsilon_{ijkl}\;∼\;N(0,\;\sigma_{\varepsilon }^{2}))$.

It should be noted that when only 1 tree was measured per plot, the residual error corresponded to the experimental error. Site-specific analyses were also carried out using the same model but without the site and site × clone interaction effects.

To determine if the variance estimates were significantly >0, 1-tailed likelihood ratio tests were performed using the same procedure (comparing models with and without the term to be tested).

### Genotypic parameters

#### Genotypic coefficient of variation

The genotypic coefficient of variation was calculated using the equation presented in Stener and Hedenberg (2003):


$$CV_{G}\;=\;\;\frac{\sigma_{C}}{\bar{Y}}\times 100,$$

where $\sigma_{C}\;$ is the square root of the clonal variance $\sigma_{C}^{2}$ and $\bar{Y}$ is the phenotypic mean.

#### Broad-sense heritability and genotypic type B correlation

Broad-sense heritability (repeatability, $H_{C}^{2}$) and genotypic type B correlation ($r_{B}$) were calculated as follows:


$$H_{C}^{2}=\;\frac{\sigma_{C}^{2}}{\sigma_{C}^{2}+\;\sigma_{S\times C\;}^{2}+\sigma_{B(S)\times C}^{2}+\;\sigma_{\varepsilon }^{2}},$$

$$r_{B}\;=\;\;\frac{\sigma_{C}^{2}}{\sigma_{C}^{2}+\;\sigma_{S\times C}^{2}}.$$

Standard errors of these parameters were obtained using the Delta method, based on Taylor series (Lynch and Walsh 1998).

Additionally, the ratio ($\sigma_{S\times C}^{2}/\sigma_{C}^{2}$) was also examined. According to Shelbourne (1972), when the value of $\sigma_{S\times C}^{2}\;$ exceeds 50% of $\sigma_{C}^{2}$, the site–clone interaction is considered to have a significant effect on the potential genetic gain from a breeding program. This threshold ratio of variance components by Shelbourne equates to a genetic correlation type B of approximately 0.67 (Ding 2008).

#### Phenotypic and genotypic correlations

Phenotypic and genotypic correlations were calculated between all pairs of variables using the MIXED and IML procedures of SAS, considering the correlation between traits *Y*_1_ and *Y*_2_ measured on the same tree. The bivariate analysis of variance model used was similar to the model presented in *Models* section, except that the trait factor was added to the model, along with all its interactions with other factors, considering the bivariate normal distribution. It should also be noted that the plot error ($\sigma_{B(S)\times C}^{2}$) was not included in the bivariate model in order to have the same model for all pairs of variables.

The type A genotypic correlation (*r_A_*) between traits *Y*_1_ and *Y*_2_ was obtained as follows:


$$r_{A}=\;\frac{\sigma_{C}(Y_{1},Y_{2})}{\sqrt{\sigma_{C\;}^{2}(Y_{1})\cdot \sigma_{C\;}^{2}(Y_{2})}},$$

where $\sigma_{C}(Y_{1},Y_{2})$ corresponds to the genotypic covariance between the 2 traits, while $\sigma_{C\;}^{2}(Y_{1})$ and $\sigma_{C\;}^{2}(Y_{2})$ represent the genotypic variance for trait *Y*_1_ and *Y*_2_, respectively.

Phenotypic correlations between traits *Y*_1_ and *Y*_2_ were obtained as follows:


$$r_{p}=\;\frac{\sigma_{C}(Y_{1},Y_{2})+\;\sigma_{S\times C}(Y_{1},Y_{2})+\;\sigma_{\varepsilon }(Y_{1},Y_{2})}{\sqrt{\left(\sigma_{C}^{2}(Y_{1})+\;\sigma_{S\times C}^{2}(Y_{1})+\;\sigma_{\varepsilon }^{2}(Y_{1}))\cdot (\sigma_{C}^{2}(Y_{2})+\;\sigma_{S\times C}^{2}(Y_{2})+\;\sigma_{\varepsilon }^{2}(Y_{2})\right)}},$$

where $\sigma_{C}(Y_{1},Y_{2})$, $\sigma_{S\times C}(Y_{1},Y_{2})$, and $\sigma_{\varepsilon }(Y_{1},Y_{2})$ correspond to the covariances between the 2 traits associated with clones, the interaction between the site and clones, and the residual error respectively, while $\sigma_{C}^{2}(Y_{t})$, $\sigma_{S\times C}^{2}(Y_{t})$, and $\sigma_{\varepsilon }^{2}(Y_{t})$ represent the variances for clones, site × clone interaction, and residual error for trait *t* (*t* = 1, 2).

Standard errors of these correlations were calculated once again using the Delta method, based on Taylor series (Lynch and Walsh 1998).

#### Genotypic value and their interclonal coefficient of variation

Genotypic values ($V_{G}$) correspond to the predicted gains for a trait during the deployment of clones, which are deviations from the mean of the clone population. These values were obtained for each clone using the model presented in *Models* section and the BLUP method of the MIXED procedure. Adjusted genotypic values ($V_{G\_adj}$) were obtained by adding the mean value of the trait under consideration, to be on the original scale of the different measurements.

For each trait in each breeding zone, the ranking of clones was obtained from the adjusted genotypic values. Due to the structure (1–2 clones per family) and the small size of the populations in this study (80–119 clones and 46–67 families; Supplementary Table 1), predictions of breeding values (additive genetic effect) were not calculated; indeed, population size is out of range for estimation of genetic parameters (e.g. Isik *et al*. 2005; Perron *et al*. 2013). Thus, the genetic gains from using the STs as parents will not be estimable.

The interclonal coefficient of variation was calculated as follows:


$$CV_{V_{G\_adj}}\;=\frac{\sigma_{V_{G\_adj}}}{\;{\bar{V}}_{G\_adj}}\;\times 100,$$

where the numerator and the denominator correspond respectively to the standard deviation and the mean of the adjusted genotypic values of each clone for the trait under consideration. The denominator is equivalent to the overall mean.

### Traits choices and clone ranking

Growth and wood quality traits are important characteristics for the black spruce breeding program. Trees evaluated at 15 years have DBH closely correlated with TH, making this trait less prioritized for growth selection at this age. Additionally, height is more under genetic control compared to DBH (e.g. Desponts *et al*. 2017). Therefore, the traits chosen for selection are height growth and acoustic velocity ($V_{dir}$). Acoustic velocity was chosen because it is weakly positively correlated with TH and wood density ($D_{\;pil}$) and less negatively correlated with DBH than dynamic stiffness ($\mathrm{Mo}E_{dir+pil}$), even though the latter trait is one of the most important wood traits and the most sought after for lumber products (e.g. Zobel and Buitjeenen 1989). Fundova *et al*. (2019) observed that acoustic velocity alone or an estimation of dynamic MoE with acoustic velocity and a constant for density produced similar results to a direct measurement of dynamic MoE that included the best indirect estimate of density. Furthermore, to maximize efficiency, Rashidi-Jouybari *et al*. (2022) also selected these 2 traits for white spruce breeding.

One of the best ways to rank trees for 2 traits is to use a SI that weights each genotypic value (*Genotypic value and their interclonal coefficient of variation* section), in order to maximize genetic gain for each trait:


$$\mathrm{SI}=w_{1}\times V_{G\_TH\_std}\;+\;w_{2}\;\times \;V_{{G\_V}_{dir\_std}},$$

where $V_{G\_TH\_std}$ and $V_{{G\_V}_{dir\_std}}$ are the standardized estimated genotypic values and $w_{1}$ and $w_{2}$ are the relative weights given to each trait ($0\leq w_{i}\leq 1$ and $w_{1}+w_{2}=1$) that maximize the overall gain for a selection of top 10 clones. For each zone, a graph of the relative genetic gain (%), calculated as the ratio of the expected gain to the maximum possible gain from single-trait selection, was then generated by varying the weights. The intersection of the 2 curves on the graph was used to determine the optimal weights (rounded to the nearest 5%).

For each breeding zone, selection differentials (*S*) were then calculated for each trait, considering selection of the top 10 clones based on the SI, firstly using values from the combined analysis of both sites and secondly for each site separately, as follows:


$$\;S\;=\;\frac{\sum_{i=1}^{10}\;V_{G\_ij}/10}{{\bar{Y}}_{j}}\;\times 100,$$

where $V_{G\_ij}$ is the genotypic value of trait *j* for the ith best clone according to the index and ${\bar{Y}}_{j}$ is the overall mean of trait *j*.

### BLUP-based stability indexes

Due to the limitation of 2 clone trials per population in the current study, stability assessment using linear regression analysis (e.g. Lynch and Walsh 1998) was not applied, although this method directly identifies genetic elements that vary with changes in the environment (e.g. Skrøppa and Steffenrem 2021). However, for populations that at least 1 trait of the SI (see above section *Traits choices and clone ranking*) showed significant G × E interaction variance component with $r_{B}$ lower or very close to the threshold ($r_{B}\;\leq 0.67$), 2 parametric BLUP-based stability indexes were calculated to assess the phenotypic stability of each clone for those traits (TH and $V_{dir}$), as genotypic values, clone ranking, and selection are based on BLUP.

First, the harmonic mean of the relative performance of genotypic values (HMRPGV), a BLUP-based stability index that considers stability, adaptability, and mean performance simultaneously for a trait, was calculated for each clone (proposed by Resende 2007 cited by Colombari Filho *et al*. 2013; Pour-Aboughadareh *et al*. 2022):


$$\mathrm{HMRPG}V_{i}=\;\frac{E}{\sum_{\;j=1}^{E}\frac{1}{V_{G\_adj\_ij}/{\bar{Y}}_{j}}},$$

where $V_{G\_adj\_ij}$, ${\bar{Y}}_{j}$, and *E* are the adjusted genotypic value (BLUP) for the clone *i* in the site *j* (see Braga *et al*. 2020), the grand mean for each site *j*, and the number of sites, respectively. For concerned traits, clone BLUPs on each site from the multisites analysis were used. This index is also used to classified genetic elements (e.g. Colombari Filho *et al*. 2013) as high values are associated to higher performance, adaptability, and stability.

Finally, to estimate the contribution of each clone to the interaction variance, a modified ecovalence value (*W*; Wricke 1962) was calculated for each of them using their genotypic values (BLUP) of a trait of interest on each site from the multisite analysis and expressed in percent (Sonesson and Eriksson 2020):


$$W_{\mathrm{BLUP}\_i}=\overset{2}{\underset{\;j=1}{\sum }}\;(V_{G\_adj\_ij}-{\bar{V}}_{G\_adj\_i.}-{\bar{Y}}_{.j}-{\bar{Y}}_{..}{)}^{2},$$

where $W_{\mathrm{BLUP}\_i}$ is the genotypic ecovalence (contribution) of clone *i*, $V_{G\_adj\_ij}$ is the adjusted genotypic value of clone *i* in environment *j*, ${\bar{V}}_{G\_adj\_i.}$ is the mean adjusted genotypic value of clone *i* across environments, ${\bar{Y}}_{.j}$ is the mean on environment *j*, and ${\bar{Y}}_{..}$ is the overall mean, that is the mean of all clones on both sites. Clones with low BLUP–ecovalence values for a trait are more stable since they have reduced deviation from the mean across environments. As *W* is linearly associated to Shukla's stability variance (Kang and Miller 1984), its statistical significance was tested with the Shukla (1972) method.

## Results

### Genotypic parameters from the combined analysis of clone trials

#### Descriptive statistics of traits

The height and diameter growth of clones in the mixed forest (zone D) are significantly higher and less variable compared to those of other populations (Supplementary Table 1). However, the values obtained in the other 2 zones and for the 3 other populations are comparable. The coefficients of variation (CVs) are relatively low for zone D, with values of 9.7 and 15.8% (Supplementary Table 1), indicating a homogeneous population (CV ≤ 15%; Ouellet 1990), whereas for the other zones, populations appear moderately heterogeneous as CV ranges between 15 and 30% (e.g. Ouellet 1990).

For wood traits, average values of $V_{dir}\;$ are similar across zones. However, notably higher values of $D_{\;pil}$ are observed in the 2 populations of zone A, indicating generally lower wood density. Average values of $\mathrm{Mo}E_{dir+pil}\;$ are comparable across zones, although slightly higher in zone C (mossy spruce). The CVs for $\mathrm{Mo}E_{dir+pil}$ are moderate (24.1–36.1%), and thus, populations are heterogeneous. However, populations are homogeneous for $V_{dir}$ and $D_{\;pil}$ with CV values ranging from 9.5 to 16.0%.

#### Genotypic parameters

The variance among clones (${\hat{\sigma }}_{C}^{2}$) remains significant and substantial for height growth in all populations (Tables 2 and 3). The proportion of variance attributable to clones for TH is higher in the eastern trials, with 27.0 and 19.3% of the total variance in zones D and C, respectively, compared to 11.4–12.8% in zone A. The variance among clones remains predominant compared to ${\hat{\sigma }}_{S\times C}^{2}$, but in A-East population, it is nearly equivalent to ${\hat{\sigma }}_{S\times C}^{2}$. Interclonal variance for DBH remains relatively low, although a significantly higher value is observed in zone D. The proportion of variance attributable to clones for DBH is stable and more significant than ${\hat{\sigma }}_{S\times C}^{2}$, especially in zone D. G × E interaction is almost negligible in zones D and C for growth traits (${\hat{r}}_{B}$ very high) but becomes more important for TH in zone A, especially for A-East population. These results suggest that site effects could significantly impact genetic gain for height in zone A populations, particularly for A-East population. The stability index (${\hat{\sigma }}_{S\times C}^{2}$/${\hat{\sigma }}_{C}^{2}$ ratio) indicates a similar trend, particularly with a very high proportion (0.88) for TH in A-East population. The value of the genotypic coefficient of variation ($CV_{G}$) is relatively low for TH and higher for DBH but the similar magnitude in all zones for both growth traits (4.9–6.4% for TH and 7.6–9.1% for DBH).

**Table 2.** Variance estimates, genotypic coefficient of variation, clonal repeatability, and type B genotypic correlation for growth and wood quality traits after 15–16 years for both populations of seed and breeding zone A.

	Seed and breeding zone/trait									
	A-West population					A-East population				
Parameter^*a*^	TH	DBH	*D*_*pil*_	*V*_*dir*_	MoE_*dir* + *pil*_	TH	DBH	*D*_*pil*_	*V*_*dir*_	MoE_*dir* + *pil*_
${\hat{\sigma }}_{c}^{2}$	813.8^*b*^ (243.7) [12.8]^*c*^	33.6^*b*^ (8.6) [14.6]	1.3824^*b*^ (0.2747) [38.8]	0.0415^*b*^ (0.0098) [39.1]	0.5184^*b*^ (0.1200) [40.4]	950.5^*b*^ (248.0) [11.4]	42.5^*b*^ (9.0) [12.3]	2.2433^*b*^ (0.3432) [38.4]	0.0423^*b*^ (0.0063) [54.5]	0.5330^*b*^ (0.0812) [48.7]
${\hat{\sigma }}_{s\times c}^{2}$	246.1^*d*^ (186.9) [3.9]	2.8 (5.4) [1.2]	0 (.) [0]	0.0191^*b*^ (0.0053) [18.0]	0.2181^*b*^ (0.0616) [17.0]	837.8^*b*^ (201.8) [10.0]	15.2^*b*^ (5.8) [4.4]	0.1215 (0.0920) [2.1]	0.0046^*b*^ (0.0013) [5.9]	0.0709^*b*^ (0.0205) [6.5]
${\hat{\sigma }}_{B(s)\times c}^{2}$	962.6^*b*^ (337.4) [15.2]	27.0^*b*^ (12.7) [11.8]	—	—	—	1,481.1^*b*^ (232.9) [17.7]	48.3^*b*^ (10.3) [13.9]	—	—	—
${\hat{\sigma }}_{\varepsilon }^{2}$	4,317.4 (345.6) [68.1]	166.7 (13.4) [72.4]	2.1848 (0.1417) [61.2]	0.0456 (0.0032) [42.9]	0.5477 (0.0385) [42.6]	5078.4 (229.7) [60.8]	240.7 (10.8) [69.4]	3.4697 (0.1408) [59.5]	0.0307 (0.0013) [39.6]	0.4900 (0.0200) [44.8]
${\hat{\sigma }}_{P}^{2}$	6,339.8	230.1	3.5672	0.1063	1.2843	8,347.9	346.6	5.8346	0.0776	1.0939
${\hat{\sigma }}_{s\times c}^{2}/{\hat{\sigma }}_{c}^{2}$	0.30	0.08	0	0.46	0.42	0.88	0.36	0.05	0.11	0.13
CV_*G*_	5.7	9.1	5.7	6.7	15.8	5.5	8.7	7.6	7.0	16.3
${\hat{H}}_{c}^{2}$	0.13 (0.04)	0.15 (0.03)	0.39 (0.05)	0.39 (0.06)	0.40 (0.06)	0.11 (0.03)	0.12 (0.02)	0.38 (0.04)	0.54 (0.04)	0.49 (0.04)
${\hat{r}}_{B}$	0.77^*b*^ (0.16)	0.92^*b*^ (0.14)	1^*b*^ (0)	0.69^*b*^ (0.09)	0.70^*b*^ (0.09)	0.53^*b*^ (0.10)	0.74^*b*^ (0.10)	0.95^*b*^ (0.04)	0.90^*b*^ (0.03)	0.88^*b*^ (0.04)

Parameter standard errors are shown in brackets.

Trait abbreviations: TH = total height growth in cm; DBH = diameter at breast height in mm; D_*pil*_ = wood density with penetration depth of the Pilodyn 6J Forest device in mm; V_*dir*_ = wave propagation velocity in km · s^-1^ with the Fiber-gen Hitman ST300; MoE_*dir+pil*_ = calculated indirect measure of the MoE in GPa.

^*a*^ ${\hat{\sigma }}_{C}^{2}$ = among-clone variance component estimate; ${\hat{\sigma }}_{S\times C}^{2}$ = site × clone interaction variance component estimate; ${\hat{\sigma }}_{B(S)\times C}^{2}$ = among-plot variance component estimate (corresponds to residual variance if single-tree plots); ${\hat{\sigma }}_{\varepsilon }^{2}$ = residual variance estimate due to variation within plots; ${\hat{\sigma }}_{P}^{2}$ = phenotypic (total) variance component = ${\hat{\sigma }}_{C}^{2}+{\hat{\sigma }}_{S\times C}^{2}+{\hat{\sigma }}_{B(S)\times C}^{2}+{\hat{\sigma }}_{\varepsilon }^{2}$; $CV_{G}\;$ = genotypic coefficient of variation; ${\hat{H}}_{c}^{2}$ = clonal heritability (repeatability); ${\hat{r}}_{B}$ = type B genotypic correlation.

^*b*^Significantly different from 0 (for) or significantly positive (for variance estimates) at the 5% significance level based on one-tailed likelihood ratio test.

^*c*^Percentage of each estimated variance component to total (phenotypic) variance.

^*d*^The value of the likelihood ratio test is 0.0625 and therefore close to the significance level of 0.05.

**Table 3.** Variance estimates, genotypic coefficient of variation, clonal repeatability, and type B genotypic correlation for growth and wood quality traits after 15–16 years for populations of seed and breeding zones C and D.

	Seed and breeding zone/trait									
	C					D				
Parameter^*a*^	TH	DBH	*D*_*pil*_	*V*_*dir*_	MoE_*dir* + *pil*_	TH	DBH	*D*_*pil*_	*V*_*dir*_	MoE_*dir* + *pil*_
${\hat{\sigma }}_{c}^{2}$	1,159.3^*b*^ (222.2) [19.3]^*c*^	23.0^*b*^ (4.5) [13.6]	1.4628^*b*^ (0.2550) [46.7]	0.0637^*b*^ (0.0103) [64.3]	1.5326^*b*^ (0.2464) [62.3]	1,118.7^*b*^ (198.8) [27.0]	78.2^*b*^ (13.6) [30.1]	1.1463^*b*^ (0.2011) [29.2]	0.0798^*b*^ (0.0119) [69.7]	1.1763^*b*^ (0.1878) [56.7]
${\hat{\sigma }}_{s\times c}^{2}$	176.5^*b*^ (84.5) [2.9]	0.6 (1.9) [0.4]	0.1069^*b*^ (0.0639) [3.4]	0.0019^*b*^ (0.0012) [1.9]	0 (.) [0]	0 (.) [0]	0 (.) [0]	0 (.) [0]	0.000003 (0.0008) [<0.1]	0.0948^*b*^ (0.0351) [4.6]
${\hat{\sigma }}_{B(s)\times c}^{2}$	1,232.7^*b*^ (152.4) [20.5]	41.8^*b*^ (4.8) [24.8]	—	—	—	—	—	—	—	—
${\hat{\sigma }}_{\varepsilon }^{2}$	3,439.9 (143.8) [57.3]	103.2 (4.3) [61.2]	1.5632 (0.0778) [49.9]	0.0336 (0.0016) [33.9]	0.9269 (0.0439) [37.7]	3,025.7 (129.2) [73.0]	181.3 (7.7) [69.9]	2.7826 (0.1211) [70.8]	0.0347 (0.0015) [30.3]	0.8021 (0.0365) [38.7]
${\hat{\sigma }}_{P}^{2}$	6,008.4	168.6	3.1328	0.0991	2.4595	4,144.3	259.5	3.9289	0.1145	2.0733
${\hat{\sigma }}_{s\times c}^{2}/{\hat{\sigma }}_{c}^{2}$	0.15	0.03	0.07	0.03	0	0	0	0	<0.01	0.08
CV_*G*_	6.4	7.6	7.5	7.6	17.9	4.9	8.5	6.5	10.6	23.4
${\hat{H}}_{c}^{2}$	0.19 (0.03)	0.14 (0.02)	0.47 (0.05)	0.64 (0.04)	0.62 (0.04)	0.27 (0.04)	0.30 (0.04)	0.29 (0.04)	0.70 (0.03)	0.57 (0.04)
${\hat{r}}_{B}$	0.87^*b*^ (0.06)	0.97^*b*^ (0.08)	0.93^*b*^ (0.04)	0.97^*b*^ (0.02)	1^*b*^ (0)	1^*b*^ (0)	1^*b*^ (0)	1^*b*^ (0)	0.99^*b*^ (0.01)	0.93^*b*^ (0.03)

Parameter standard errors are shown in brackets.

See Table 2 for the definition of trait abbreviations.

^*a*^  ${\hat{\sigma }}_{c}^{2}=$ among-clone variance component estimate; ${\hat{\sigma }}_{s\times c}^{2}=$ site × clone interaction variance component estimate; ${\hat{\sigma }}_{B(s)\times c}^{2}=$ among-plot variance component estimate (corresponds to residual variance if single-tree plots); ${\hat{\sigma }}_{\varepsilon }^{2}=$ residual variance estimate due to variation within plots; ${\hat{\sigma }}_{P}^{2}=$ phenotypic (total) variance component $={\hat{\sigma }}_{c}^{2}+{\hat{\sigma }}_{s\times c}^{2}+{\hat{\sigma }}_{B(s)\times c}^{2}+{\hat{\sigma }}_{\varepsilon }^{2}$; *CV*_*G*_ = genotypic coefficient of variation; ${\hat{H}}_{c}^{2}=$ clonal heritability (repeatability); ${\hat{r}}_{B}=$ type B genotypic correlation.

^*b*^ Significantly different from 0 (for ${\hat{r}}_{B}$) or significantly positive (for variance estimates) at the 5% significance level based on 1-tailed likelihood ratio test.

^*c*^ Percentage of each estimated variance component to total (phenotypic) variance.

The values obtained for wood traits are more stable compared to growth traits. We observe larger interclonal variations (${\hat{\sigma }}_{C}^{2}$) with $V_{dir}\;$ and $\mathrm{Mo}E_{dir+pil}\;$ and minimal for $D_{\;pil}$ across all regions. The proportion of variance among clones (${\hat{\sigma }}_{C}^{2}$) remains predominant and significant in all zones. However, lower values are noted for $V_{dir}\;$ and $\mathrm{Mo}E_{dir+pil}\;$ in zone A. For $D_{\;pil}$, the variance ${\hat{\sigma }}_{S\times C}^{2}\;$ remains null or very low in all zones. G × E interaction remains weak or almost nonexistent for all wood traits in all zones (${\hat{r}}_{B}$ very high) but is more pronounced in A-West population for $V_{dir}\;$ and $\mathrm{Mo}E_{dir+pil}$, indicating that the effectiveness of selection and genetic gain for these traits in this population would be diminished. The genotypic coefficient of variation $CV_{G}$ is much higher and variable according to the area for $\mathrm{Mo}E_{dir+pil}$ (15.8–23.4%) than for $D_{\;pil}$ (5.7–7.6%) and $V_{dir}$ (6.7–10.6%).

In addition, for all clone populations, rank correlations between the clone genotypic values predicted in each of the 2 clone trials indicate the same trends as G × E and ${\hat{\sigma }}_{S\times C}^{2}$/${\hat{\sigma }}_{C}^{2}$ ratio for both growth and wood traits (Supplementary Table 3).

#### Clonal heritability (repeatability)

The clonal heritability ${\hat{H}}_{c}^{2}\;$ for growth traits is generally low, with values below 0.20, except for the mixed forest zone (zone D) where it is moderate (0.27 and 0.30).

For wood quality traits, the heritability ${\hat{H}}_{c}^{2}$ ranges from moderate to high, with maximum values for $V_{dir}$ and $\mathrm{Mo}E_{dir+pil}$ around 0.4–0.7. The values for $D_{\;pil}\;$ are relatively consistent across zones (0.29–0.47), with a lower value observed for the population in zone D (0.29).

#### Phenotypic and genotypic correlations between traits

As expected, TH and DBH are highly correlated in all zones, although the value is slightly lower in the mixed forest (zone D: 0.65 vs 0.83–0.85) (Supplementary Table 2).

TH is very weakly correlated with $V_{dir}\;$ and $\mathrm{Mo}E_{dir+pil}$, sometimes negatively with the latter but always positively with $\;V_{dir}$. Phenotypic correlations are slightly higher and positive with $D_{\;pil}$ in zone C (0.32) and A-East population (0.36). The correlations between DBH and $V_{dir}$ and $\mathrm{Mo}E_{dir+pil}$ are weak to moderate and negative (wood stiffness decreases with increasing DBH). The correlations between DBH and $D_{\;pil}\;$ are moderate and positive (density decreases with increasing DBH) with a maximum value for A-East population (0.59). Between $V_{dir}$ and $D_{\;pil}$, the values are very weakly negatively correlated.

The type A genotypic correlations between TH and DBH remain high with a slightly lower value in zone D (Supplementary Table 2). The type A genotypic correlations between TH and wood traits are generally weak to moderate and positive, while those between DBH and wood traits are overall moderate and negative. However, there is a particularly high correlation between DBH and $D_{\;pil}$ for A-East population (0.64). The correlations between $V_{dir}$ and $D_{\;pil}$ are weak and negative.

### Ranking results and parent selection

TH and $V_{dir}$ weights in the SI were respectively 0.50–0.50 for zones A-West and C and 0.55–0.45 for A-East and D zones (Supplementary Fig. 1). The clones were ranked based on the index values obtained from the genotypic values of the combined test analysis or site-specific analyses for each breeding zone. By selecting the top 10 clones for each zone based on the index values from the combined analyses, we observe an overall relatively low selection differential for TH and relatively high for $\mathrm{Mo}E_{dir+pil}$ and $V_{dir}$ across all populations, while maintaining $D_{\;pil}$ values around the average of their respective populations with slight gains or losses and a small gain for DBH in all populations but lower in zone D (Table 4). The selection differential for TH is maximal in zone C (8.2%) and similar in other populations (5.2–6.8%). For $\mathrm{Mo}E_{dir+pil}$, the gain is notably higher in zone D (30.0%) compared to the other 3 populations (12.6–20.7%). The same trend is observed for $V_{dir}$.

**Table 4.** Selection differentials (BLUP %) for a selection of the 10 best clones ranked according to the index ($\mathrm{SI}=\;0.5\times V_{G\_TH\_std}+$  $0.5\;\times \;V_{{G\_V}_{dir}\_std}$ for population of zones A-West and C, and $\mathrm{SI}=\;0.55\times V_{G\_TH\_std}+0.45\;\times \;V_{{G\_V}_{dir}\_std}$ for population of zones A-East and D) for combined and individual test analyses.

	Seed and breeding zone/site											
	A-West population			A-East population			C			D		
Traits	VIL51905	DUP52005	Both sites	VIL47003	DUP46903	Both sites	RRO41401	CHV41501	Both sites	ROB34899	ASS35199	Both sites
TH	8.9	2.8	5.9	7.4	10.3	5.2	7.8	7.0	8.2	5.0	5.4	6.8
DBH	7.9	4.2	5.7	3.5	6.0	3.0	3.3	4.2	3.6	−2.7	2.4	2.2
*D*_*pil*_	−0.6	−0.7	−1.5	−0.6	−1.2	0.7	−0.1	−1.2	−1.1	−1.1	−1.1	−1.3
*V*_*dir*_	6.8	6.1	5.3	6.5	11.6	9.5	9.1	10.4	9.7	16.4	15.9	14.9
MoE_*dir*+*pil*_	14.7	13.0	12.6	13.2	24.9	17.7	17.8	21.4	20.7	34.7	33.2	30.0

See Table 2 for the definition of trait abbreviations.

In comparison with individual site selections, we find that the differential for $\mathrm{Mo}E_{dir+pil}$ obtained from the combined selection is sometimes midway between those from individual site selections (A-East population and zone C), sometimes lower (A-West population and zone D). However, the differential obtained from the combined selection for TH is higher than that from individual site selections in zones C and D but lower than the 2 individual tests for zone A-East and midway for zone A-West.

### BLUP-based stability indexes

**Fig. 2.**
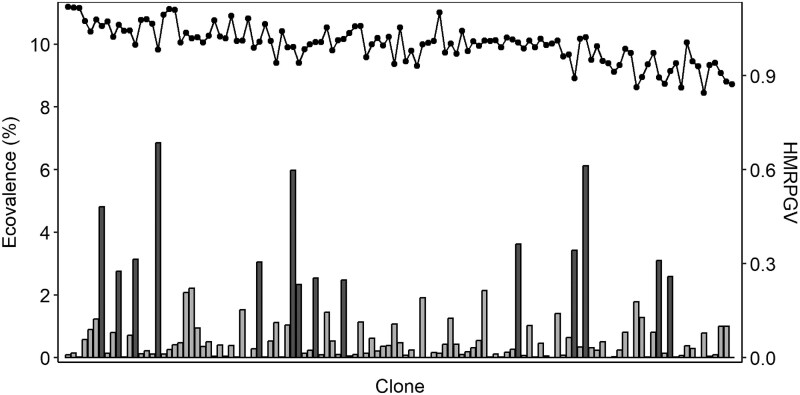
HMRPGV values (right axis and segmented line) and BLUP–ecovalence (%) (left axis and bars) of each clone for TH in the A-East population (${\hat{r}}_{B}=0.53\;\pm 0.10$; Table 2). Clones are ordered according to their SI value, the highest values (best clones according to SI) on the left. Clones that contribute significantly (*P* < 0.05) to the site–clone interaction variance are represented by dark-gray bars.

**Fig. 3.**
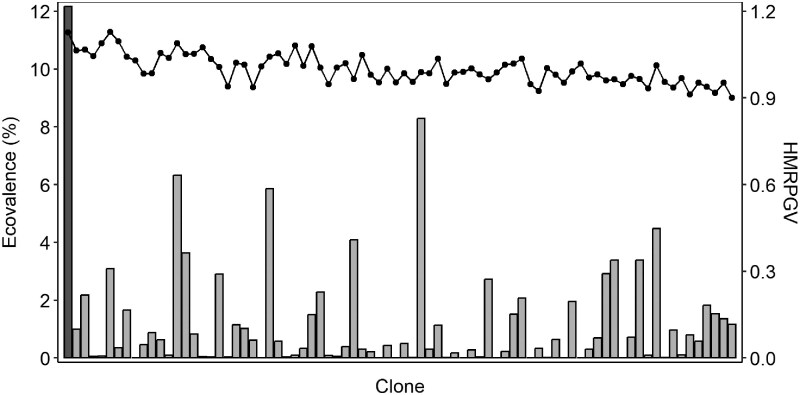
HMRPGV values (right axis and segmented line) and BLUP–ecovalence (%) (left axis and bars) of each clone for TH in the A-West population (${\hat{r}}_{B}=0.77\;\pm 0.16$; Table 2). Clones are ordered according to their SI value, the highest values (best clones according to SI) on the left. Clones that contribute significantly (*P* < 0.05) to the site–clone interaction variance are represented by dark-gray bars.

BLUP-based stability indexes were calculated for TH and $V_{dir}$ in A-East and A-West populations (see Tables 2 and 3). Figures 2 and 3 present HMRPGV values and BLUP–ecovalence (%) of each clone for TH in both populations. The top 10 clones ranked by TH adjusted genotypic mean from multisite analyses match almost perfectly with the top 10 clones ranked by HMRPGV (percentage of coincidence of 100% for A-West population and 90% for A-East population), but did not coincide with the top 10 ranked by the BLUP–ecovalence (%) (0 out of 10 in common for both A-West and A-East populations) (see Figs. 2 and 3). The match of the top-ranked 10 clones based on SI (first 10 clones on the left of Figs. 2 and 3) fit also better with the top-ranked 10 clones of HMRPGV (6 and 4 in common in the A-West and A-East populations, respectively) than BLUP–ecovalence (1 and 0 out of 10 in common in the A-West and A-East, respectively). There is only 1 clone that contributes significantly to the TH G × E interaction in the A-West population, whereas there are 14 clones in the A-East population.

In both populations, we observed for $V_{dir}$ that few individual clones were not stable for that trait (data not shown).

## Discussion

In the context of climate change and environmental instability, the phenotypic plasticity of trees is crucial information in reforestation programs. To our knowledge, this is the first study that illustrates high individual stability directly for seed orchard parents. Cutting black spruce was still possible for several individuals at the tree selection age in progeny trials in the boreal forest of Québec, at least in quantities sufficient for establishing clone trials. Consequently, forest geneticists have established such trials following the selection of parents for the next breeding cycle of the genetic improvement program. This has allowed for the study of genotypic parameters of growth and wood traits and, importantly, the characterization of the phenotypic stability of future parents, which is generally high.

The 2 clone trials per population (80–119 clones) are located on either side of a seed and breeding zone, under contrasting climates and pedoclimatic conditions, providing the opportunity to specify the G × E interaction of certain growth and wood traits for clones. It was already known that black spruce families could demonstrate significant phenotypic plasticity for growth and wood traits depending on the environment (e.g. Perron *et al*. 2013; Desponts *et al*. 2017), but few studies have evaluated the maintenance of relative performance of individuals compared to their population for economically important traits (e.g. Mullin and Park 1994). The comparative planting sites in this study allowed to evaluate how different genotypes maintained their performance across areas with very different conditions. As expected, growth was faster in eastern yellow birch-balsam fir bioclimatic domains (zone D), where pedoclimatic conditions are more favorable. However, the CVs for growth traits remain comparable and relatively low or moderately high in all zones. Nonetheless, the average phenotypic values of wood parameters vary little from one zone to another, and CVs remain low for $D_{\;pil}$ and $V_{dir}\;$ and moderate for $\mathrm{Mo}E_{dir+pil}$.

For growth traits, the variance is mostly associated with clones for all populations. This observation is similar to what has been observed for other populations and species of spruces (e.g. Mullin and Park 1994; Skrøppa and Steffenrem 2021), as well as other conifers (e.g. Baltunis *et al*. 2013; Pan *et al*. 2020), indicating significant interclonal variations. The genotypic heritability is low to moderate for growth, as found by Mullin and Park (1994) for this species. However, they observed a relatively low G × E effect, likely attributable to the population and more similar climate and pedoclimatic conditions over a limited territory, compared to the situation in several distinct zones in the present study. In our seed and breeding zones, the G × E interaction effect is also very low in zone D and low in zone C, even though the sites in zone C differ more: one site is further south with a more temperate climate and particularly sandy soil and the other more boreal with richer soil. However, a higher G × E interaction is noted for TH in populations of zone A, especially for A-East population, while it is almost nil in the other 2 zones. Indeed, the genotypic parameters (${\hat{r}}_{B}$ and ${\hat{\sigma }}_{S\times C}^{2}$/${\hat{\sigma }}_{C}^{2}$) (Tables 2 and 3) clearly reflect the differences in clone performance under very contrasting pedoclimatic conditions between tests in zone A (Table 1). The G × E interaction observed for TH in A-East population, using the ${\hat{r}}_{B}\;$ value, is well below the 0.67 threshold at which 2 distinct improvement populations or deployment zones should be favored (Ding 2008). With sites highly differentiated by both climate and soil, an effect of the site is observed for this population, which could diminish the validity of clone ranking by combined site analysis and reduce the expected genetic gains following selection in A-East population using combined analysis (2 sites and overall genotype rank). It is difficult to attribute this site effect to a single environmental factor, but the heavy clay of the Duparquet site seems to be an important distinguishing element of the height growth performance of some clones in this population, and to a lesser extent, A-West population, given that many climatic variables are not too dissimilar between the 2 sites (Table 1). As clay soil tends to be very compacted and has a high-water retention capacity, it could have had an impact on growth variation. In a previous study, the site effect for a northern population was attributed to soil fertility (Desponts *et al*. 2017), which seems less evident in the present study. The results suggest that the highly contrasting conditions within zone A, such as a more continental climate and clay soils in Duparquet, constitute too great a contrast compared to the eastern site of the zone (Villiers; Fig. 1), and individualized selections should be considered, at least for growth within this vast zone covering over 5° of longitude.

For wood traits, genotypic heritabilities remain moderate to high in all populations. The genotypic parameters of wood properties are more stable and generally indicate a very low site effect. The G × E effect remains almost null everywhere except for $V_{dir}$ and $\mathrm{Mo}E_{dir+pil}$ in population A-West, which in this specific case could be attributed to a smaller sample size compared to other populations. $V_{dir}$ measurements are more variable from tree to tree due to the presence of knots, curvature, etc., and biases may have occurred in the average of certain clones with too small a sample size at 1 site (Duparquet). The genotypic parameters observed for wood traits correspond to what is generally reported for conifers (e.g. Hannrup *et al*. 2004).

The phenotypic and genotypic correlations between growth parameters, particularly TH, and wood properties are generally low and positive, allowing for selection based on 2 parameters (TH and $V_{dir}$) without significantly reducing genetic gains compared to selection for a single trait. Only small losses are observed for DBH, which is not a selection character at this juvenile stage. For all populations, the maximum selection differential is obtained for $\mathrm{Mo}E_{dir+pil}$, with a limited gain for TH and a density that is maintained. The lower gains obtained for TH are explained by the fact that these clones come from a previous intense selection for this trait (about 0.2% or 1,200 ST retained from 540,000 candidates-trees—see Desponts and Numainville 2013 for more details). Therefore, operational rankings in the black spruce genetic improvement program will be based on an index including TH and $V_{dir}$ (e.g. Desponts 2019).

Several studies have shown that growth is more sensitive to changes in environmental conditions compared to the mechanical properties of wood, particularly for clonal populations of *Picea abies* (Nguyen *et al*. 2022), *Larix kaempferi* (Pan *et al*. 2020), and *Pinus taeda* (Antony *et al*. 2014), as well as with half or full-sibs progeny tests for *P. mariana* (Desponts *et al*. 2017; Lenz *et al*. 2017). As illustrated previously (with ${\hat{r}}_{B}$ and ${\hat{\sigma }}_{S\times C}^{2}$/${\hat{\sigma }}_{C}^{2}$), this increased sensitivity of growth is more pronounced among clones of populations in zone A with tests located in particularly differentiated environmental conditions. Therefore, 2 BLUP-stability indexes were calculated to characterize each clone. Additionally, for those populations, the comparison of the individual site ranking of the top 10 clones with the ranking combining 2 sites was more variables than for other populations (Table 4). In those zone A populations, the clones ranking with HMRPGV for TH are well in line with the SI ranking (Figs. 2 and 3). Moreover, these populations included many genotypes that are both highly performant and very stable, but also individuals whose growth performance relative to their population differs from one site to another. In population A-East, for TH growth, significant BLUP–ecovalence values appear in 14 clones and two of the top 10 rankings based on SI, suggesting that selection should be more regional rather than at the breeding zone scale to ensure its validity and maximize predicted genetic gains. Effectively, these results indicated that the phenotypic stability of many clones is low on both sides of this zone, suggesting a need to revise the deployment territory of this population, at least for the clay soils area. In this case, the results with 2 sites are not sufficient to precisely determine which factor would be predominant in the yield instability of many clones, so more tests would be needed. As we could see in Figs. 2 and 3, the BLUP–ecovalence values are particularly interesting to document stability of the individuals, even in a population without significant G × E effect (TH in A-West population). We deliberately did not use a multitrait stability index since we wanted to clearly see the stability of each ST for every single trait. Indeed, one of the disadvantages of classification tools such as SI, and probably also of multitrait stability indices, is that they poorly classified individuals and masked those that are highly unbalanced between the classification of each trait. For example, in Figs. 2 and 3, HMRPGV combining information of performance and stability on 1 trait misclassified many clones according to their stability only. Effectively, as compared to the genotypic ecovalence that characterizes only stability (Figs. 2 and 3), many good ranking clones with HMRPGV present high BLUP–ecovalence values. In the context of climate change and increasingly unstable conditions, this adaptability for phenotypic stability should be considered an important selection criterion (e.g. Mátyás 2006; Joyce and Rehfeldt 2017). The BLUP-stability index analysis that we used also highlights that for estimating stability of genetic elements, we can benefit of the BLUP properties by directly characterizing the stability of genotypic or breeding values and conducting only 1 type of linear mixed model, as compared to many other stability indices that require additional analysis and even additional software (Pour-Aboughadareh *et al*. 2022).

Initially, the 10-year height growth results (not illustrated in the present study) guided the choice of parents for the first breeding cycle of control crosses in the A, B, C, and D zones. Subsequently, the present 15-year results guided selective harvest recommendations in Québec black spruce clonal seed orchards (SO-2). Currently, across all zones, the clones serve as parents for the next generation, and the results obtained guide us in selecting clones demonstrating high stability for desired traits. It would be conceivable, as suggested by de la Mata *et al*. (2012), to eliminate the least stable clones to reduce the G × E effect in these populations, although this could reduce their genetic diversity. Nevertheless, it is particularly interesting to note that high-performing genotypes demonstrating broader adaptation to environmental conditions can be identified for both growth and wood properties in all populations.

## Conclusion

The study of clone trials has provided new insights into the genotypic parameters and clonal stability within contrasting environments for STs constituting the Québec's black spruce improvement populations across 3 seed and breeding zones. Genotypic parameters, particularly G × E effects, on growth and wood quality traits were estimated, demonstrating that the environmental effect on gene expression is generally stable.

The study also shows that BLUP-based stability indexes could decipher G × E and identify unstable clones, especially in zone A, which covers a vast territory with more contrasting pedoclimatic conditions. Moreover, the results illustrate that in most cases under contrasting environments, black spruce individuals exhibit stable performance for growth and wood traits. This provides a solid foundation for continuing the identification and selection of high-performing, adapted, and resilient genetic elements in a context of global change.

## Supplementary Material

jkaf120_Supplementary_Data

## Data Availability

The datasets and scripts generated during and/or analyzed during the current study are freely available in the open access at Federated Research Data Repository. Recommended citation for this dataset: Perron, M. (2025). Black spruce phenotypic data and computed variables from clone trials. https://doi.org/10.20383/103.01219. All measurements were taken after the end of a growing season. The present public domain data deposition in Digital research Alliance of Canada does not exclude our right to grant a license to share information or biological material with a third party, according to the Intellectual Property Policy (IPP) of our organization (Direction de la recherche forestière du Ministère des Ressources naturelles et des Forêts, Gouvernement du Québec), which is in line with Québec and Canadian government laws concerning biological material as well as plant breeders’ rights, so parents and families were properly anonymized in all the present databases. The new identifiers have been added to the original databases that are deposited in our in-house genetic database. Supplemental material available at G3 online.
