# Supplementary material for: Mainly high phenotypic stability of black spruce clones for growth and wood traits in contrasted environments within the current breeding zones and multitrait selection in Québec's seed and breeding zones
Source: G3 (Bethesda). 2025 May 30;15(9):jkaf120. doi: 10.1093/g3journal/jkaf120 (PMC12405895; doi:10.1093/g3journal/jkaf120)

Supplementary Figure 1. Relative gain as function of variation in the weight of total height (TH) for a top-ranked 10 clones selection. The optimal selection index (weights rounded to the nearest 5%) corresponds to $SI = 0.5 \times V_{G\_TH\_std} + 0.5 \times V_{G\_V_{dir}\_std}$ for population of zones A-West and C, and $SI = 0.55 \times V_{G\_TH\_std} + 0.45 \times V_{G\_V_{dir}\_std}$ for population of zones A-East and D.

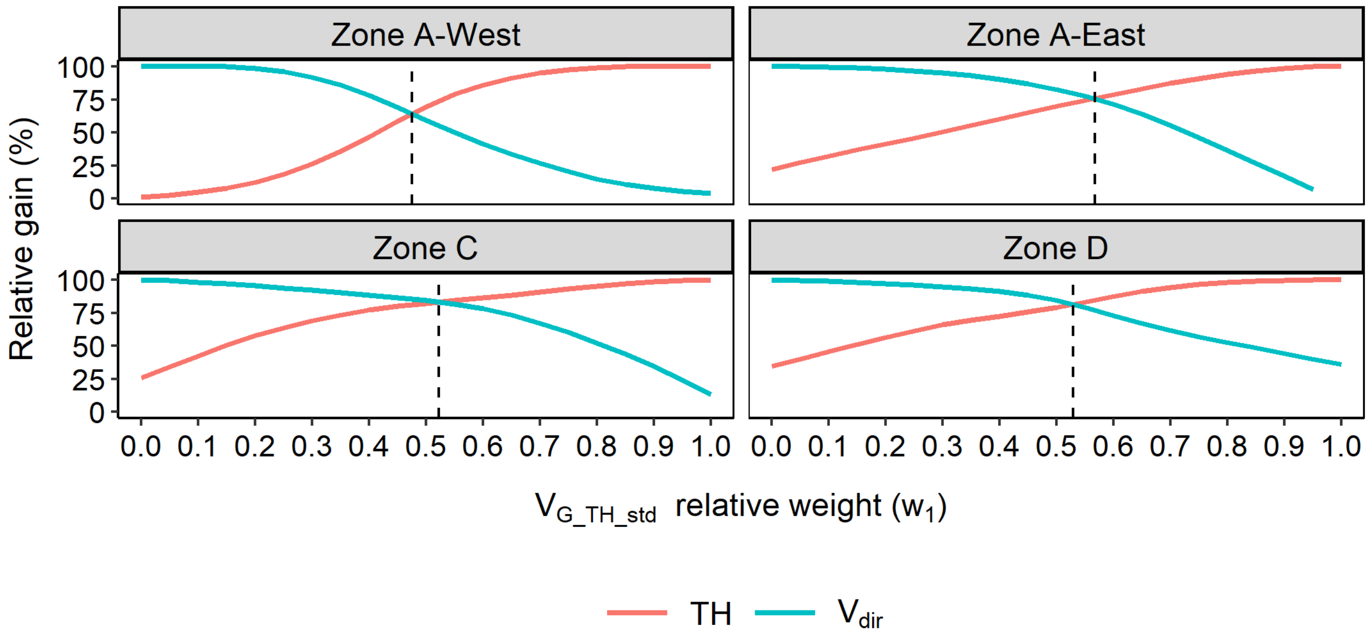

Supplement: jkaf120_Supplementary_Data [file jkaf120_supplementary_data.zip › Supplementary_Figure_1_jkaf120.pdf]
