# Supplementary material for: Mainly high phenotypic stability of black spruce clones for growth and wood traits in contrasted environments within the current breeding zones and multitrait selection in Québec's seed and breeding zones
Source: G3 (Bethesda). 2025 May 30;15(9):jkaf120. doi: 10.1093/g3journal/jkaf120 (PMC12405895; doi:10.1093/g3journal/jkaf120)
Supplement: jkaf120_Supplementary_Data [file jkaf120_supplementary_data.zip › Supplementary_Table_1_jkaf120.pdf]

Supplementary Table 1. Mean and coefficient of variation (CV (%), in brackets) of growth and wood quality traits measured on 15-16 year-old black spruce at all sites

Seed and breeding zone	A West population	A East population	C	D
Number of trees (growth traits – wood quality traits)	1122 – 572	2731 – 1441	2723 – 998	1214 – 1173
Number of clones	80	119	88	98
Number of famillies	46	63	51	67
Number of ramet/clone (growth traits – wood quality traits)	14.0 – 7.2	22.9 – 12.1	30.9 – 11.3	12.4 – 12.0
TH (cm)	499.6 (19.3)	557.0 (19.7)	532.7 (17.5)	684.9 (9.7)
DBH (mm)	63.7 (25.5)	75.1 (25.1)	63.3 (23.6)	103.8 (15.8)
D_{pil} (mm)	20.7 (9.5)	19.8 (12.7)	16.1 (12.3)	16.5 (16.0)
V_{dir} (km/s)	3.0 (13.8)	2.9 (9.9)	3.3 (11.0)	2.7 (13.3)
$MoE_{dir+pil}$ (GPa)	4.6 (29.6)	4.5 (24.1)	6.9 (24.8)	4.6 (36.1)
