# Supplementary material for: Mainly high phenotypic stability of black spruce clones for growth and wood traits in contrasted environments within the current breeding zones and multitrait selection in Québec's seed and breeding zones
Source: G3 (Bethesda). 2025 May 30;15(9):jkaf120. doi: 10.1093/g3journal/jkaf120 (PMC12405895; doi:10.1093/g3journal/jkaf120)
Supplement: jkaf120_Supplementary_Data [file jkaf120_supplementary_data.zip › Supplementary_Table_2_jkaf120.pdf]

Supplementary Table 2. Phenotypic and genotypic correlations (standard errors in brackets) between different growth and wood quality traits for the 4 clone trial series.

	Phenotypic correlations				Genotypic correlations			
Traits	A-West population	A-East population	C	D	A-West population	A-East population	C	D
TH - DBH	0.83 (0.01)	0.84 (0.01)	0.85 (0.01)	0.65 (0.02)	0.68 (0.10)	0.72 (0.07)	0.69 (0.07)	0.56 (0.09)
TH - V_{dir}	< 0.01 (0.06)	0.09 (0.04)	0.06 (0.05)	0.15 (0.05)	0.19 (0.18)	0.03 (0.14)	0.28 (0.12)	0.27 (0.11)
TH - D_{pil}	0.05 (0.06)	0.36 (0.03)	0.32 (0.05)	0.14 (0.04)	0.04 (0.17)	0.32 (0.13)	0.09 (0.13)	-0.07 (0.13)
TH - $MoE_{dir+pil}$	-0.04 (0.06)	-0.13 (0.04)	-0.08 (0.05)	0.07 (0.05)	0.12 (0.18)	-0.09 (0.11)	0.20 (0.12)	0.24 (0.11)
DBH - V_{dir}	-0.28 (0.05)	-0.11 (0.04)	-0.15 (0.05)	-0.25 (0.05)	-0.40 (0.15)	-0.18 (0.12)	-0.09 (0.13)	-0.32 (0.10)
DBH - D_{pil}	0.33 (0.05)	0.59 (0.02)	0.41 (0.04)	0.26 (0.04)	0.35 (0.14)	0.64 (0.08)	0.26 (0.12)	0.18 (0.12)
DBH - $MoE_{dir+pil}$	-0.36 (0.05)	-0.40 (0.03)	-0.29 (0.04)	-0.30 (0.05)	-0.48 (0.14)	-0.43 (0.11)	-0.21 (0.12)	-0.38 (0.10)
V_{dir} - D_{pil}	-0.07 (0.06)	-0.11 (0.05)	-0.07 (0.07)	-0.16 (0.05)	-0.18 (0.15)	-0.13 (0.10)	-0.10 (0.12)	-0.25 (0.11)
V_{dir} - $MoE_{dir+pil}$	0.92 (0.01)	0.85 (0.01)	0.86 (0.02)	0.88 (0.01)	0.92 (0.02)	0.90 (0.02)	0.90 (0.02)	0.97 (0.01)
D_{pil} - $MoE_{dir+pil}$	-0.44 (0.05)	-0.59 (0.03)	-0.54 (0.05)	-0.54 (0.04)	-0.55 (0.11)	-0.54 (0.08)	-0.51 (0.09)	-0.51 (0.09)
