# Supplementary material for: Mainly high phenotypic stability of black spruce clones for growth and wood traits in contrasted environments within the current breeding zones and multitrait selection in Québec's seed and breeding zones
Source: G3 (Bethesda). 2025 May 30;15(9):jkaf120. doi: 10.1093/g3journal/jkaf120 (PMC12405895; doi:10.1093/g3journal/jkaf120)
Supplement: jkaf120_Supplementary_Data [file jkaf120_supplementary_data.zip › Supplementary_Table_3_jkaf120.docx]

Supplementary Table 3. Spearman correlation coefficients (rank correlation) between clone genotypic values obtained for each of the two clone trials for each clone populations for growth and wood quality traits.

| Trait | Breeding zone / Sites | | | |
| --- | --- | --- | --- | --- |
|  | A-West population | A-East population | C | D |
|  | VIL51905 vs DUP52005 | VIL47003 vs DUP46903 | RRO41401 vs CHV41501 | ROB34899 vs ASS31599 |
| HT | 0.47 | 0.37 | 0.70 | 0.67 |
| DBH | 0.37 | 0.43 | 0.60 | 0.66 |
| D*_pil_* | 0.67 | 0.73 | 0.70 | 0.76 |
| V*_dir_* | 0.58 | 0.80 | 0.89 | 0.93 |
| MoE*_dir+pil_* | 0.59 | 0.77 | 0.89 | 0.92 |
| $SI=w_{1}\times V_{G\_TH\_std} + w_{2} \times V_{G\_V_{dir\_std}}$* | 0.64 | 0.52 | 0.83 | 0.83 |

*See Figure S1 for value of weights $w_{1}$ and $w_{2}$ in a specific population
